# Enhanced phosphorus availability and uptake in *Salvia miltiorrhiza* associated with humic-induced changes in soil phosphorus fractions

**DOI:** 10.1038/s41598-026-54576-z

**Published:** 2026-05-26

**Authors:** Xue Mi, Xinmeng Yang, Yue Li, Xinjun Wang, Yongping Zhao, Aigen Fu, Zhe Wang

**Affiliations:** 1 Chinese Education Ministry’s Key Laboratory of Western Resources and Modern Biotechnology, Key Laboratory of Biotechnology, College of Life Sciences, Northwest University, Taibei North Road, #229, Beilin district, Shaanxi Province 710069 Xi’an, China; 2 School of Biomedical and Food Engineering, Shangluo College, North New Road, #10, 726000 Shangluo, China

**Keywords:** Humic acid, Phosphorus fractions, Phosphorus transformation, Phosphorus-solubilizing microorganisms, *Salvia miltiorrhiza*, Biotechnology, Environmental sciences, Microbiology, Plant sciences

## Abstract

Humic acid (HA) is known to improve phosphorus (P) availability in agricultural soils. Yet, the underlying mechanisms by which it influences the microbial community and subsequent P turnover remain unclear. In this study, a pot experiment was conducted using soil from a three-year *Salvia miltiorrhiza* (*S. miltiorrhiza*) cultivation system, in which the soil was amended with three HA concentrations (T1: 100-fold dilution, T2: 200-fold, T3: 400-fold) alongside an untreated control (CK). We determined P uptake by *S. miltiorrhiza*, soil P fractions, phosphatase activities, along with high-throughput sequencing of the microbial communities to specifically target those associated with P transformation. The results showed that HA application significantly enhanced root P uptake, with increases of 68.59% and 91.05% under T2 and T3, respectively. Consequently, soil Olsen-P content decreased by 19.19% and 15.20%, respectively, consistent with the depletion of available P under enhanced plant uptake. Soil P fractionation further revealed that HA application decreased inorganic P by 20.91% and 32.29%, respectively, under T2 and T3 treatments. Specifically, H_2_O-P decreased by 62.1% and 73.61%, and NaHCO_3_-Pi decreased by 53.21% and 50.48%, respectively, under the T2 and T3 treatments. In parallel, acid phosphatase activity was increased by 68.72% and 66.67% under the T2 and T3 treatments compared to CK. Comparative high-throughput sequencing between T2 and CK revealed that HA application enriched key microbial genera associated with P cycling, including *Sphingomonas*, *Nitrospira*, *Ferruginibacter*, and *Hyphomicrobium*. Collectively, these findings suggest that HA may promote P mobilization and mineralization through association with microbial communities, thereby potentially enhancing P bioavailability and plant uptake. These findings offer new perspectives on the associations between HA application, microbial community shifts, and P use efficiency, suggesting a potential approach that merits further evaluation for P management in agricultural systems.

## Introduction

*Salvia miltiorrhiza* Bunge (*S. miltiorrhiza*) is a commercially important medicinal plant in China, valued for its dried roots, which are widely used to treat cardiovascular and cerebrovascular diseases, hypertension, and ischemic stroke 1^3^. Due to the scarcity of wild resources and high market demand, its cultivation has expanded rapidly^4^^5^. However, intensive cultivation practices coupled with suboptimal fertilizer management often lead to reduced yield and quality of *S.miltiorrhiza*, largely due to declining soil fertility, biodiversity loss, and the accumulation of soil-borne pathogens^6,7^. Therefore, developing scientifically sound fertilization strategies is essential to enhance both the productivity and quality of *S. miltiorrhiza*.

Phosphorus (P) is an essential macronutrient and a key component of nucleic acids, phospholipids, and adenosine triphosphate (ATP), supporting plant growth, metabolism, and energy transfer^8,9^. Adequate P fertilization is critical for dry matter accumulation and bioactive compound synthesis in *S. militorrhiza*^10,11^. Phosphorus availability also influences plant disease resistance, while excessive P may increase pathogen susceptibility, adequate P generally promotes defense through beneficial microbial interactions^12^^13^. However, plant-available P is often limited in agricultural soils due to chemical fixation and intensive cropping^14^. Thus, improving the bioavailability is essential for sustainable *S. miltiorrhiza* production.

Soil P exists in various fractions, broadly categorized as labile-P (Resin-P, NaHCO_3_-Pi, NaHCO_3_-Po), moderately available-P (NaOH-Pi, NaOH-Po), and stable-P pools (HCl-P, Residual-P). The conversion of stable P into plant-available forms is essential for maintaining soil P fertility. Microorganisms facilitate P cycling through the secretion of phosphatases that mineralize organic P (Po) into inorganic orthophosphate (Pi), thereby enhancing soil P availability^15^. Soil microbial communities also contribute to nutrient cycling through the decomposition of plant litter, thereby coupling with plant and soil P dynamics^16^. Among these microbes, P-solubilizing bacteria (PSB) represent a key functional group capable of converting insoluble P into bioavailable forms^17^. Field studies consistently demonstrate the potential of PSB to enhance P uptake and crop yields across diverse agricultural systems^18^. Critically, the microbial mediation of soil P-cycling is governed by the production of key enzymes such as phosphatases, which facilitate organic P mineralization, along with other mechanisms involved in inorganic P solubilization and transporter^19,20^. Understanding the abundance and activity of these microbial drivers provides a mechanistic basis for evaluating P turnover dynamics and bioavailability in the rhizosphere.

Humic acids (HA) are major components of soil organic matter and play a pivotal role in promoting soil health and plant growth^21^. As such, HA-based products have emerged as a promising strategy for sustainable agriculture^22^. HA stimulates plant growth directly by modulating phytohormone production (e.g., auxin and cytokinin) and enhancing metabolic enzyme activity^23,24^. Indirectly, it improves soil fertility and nutrient availability through chelation and co-transport mechanisms^25,26^. Notably, HA has been shown to enhance the efficiency of sparingly soluble P fertilizers by increasing P solubility and improving plant P utilization^27,28^. In addition, HA application can reshape soil microbial communities, increasing the diversity and abundance of bacteria and fungi, particularly taxa associated with P solubilization and mineralization^29,30^.

Conventional cultivation of *S. Miltiorrhiza* requires a 2–3 year growth cycle followed by a 1–2 year fallow period to mitigate soil degradation from continuous monocropping^31^. However, phosphorus (P) availability often remains limited in soils subjected to such systems. We hypothesized that humic acid (HA) amendment could alleviate this limitation by altering soil P fractions and reshaping the microbial community associated with P cycling. To test this hypothesis, we assessed HA-induced changes in soil P fractions and microbial community composition, and evaluated their implications for plant P uptake. This study provides a theoretical basis for integrating HA into fertilization strategies to enhance *S. Miltiorrhiza* production and promote sustainable agricultural practices.

## Results

### Effects of HA on dry matter accumulation and P uptake of S.miltiorrhiza

**Fig. 1:**
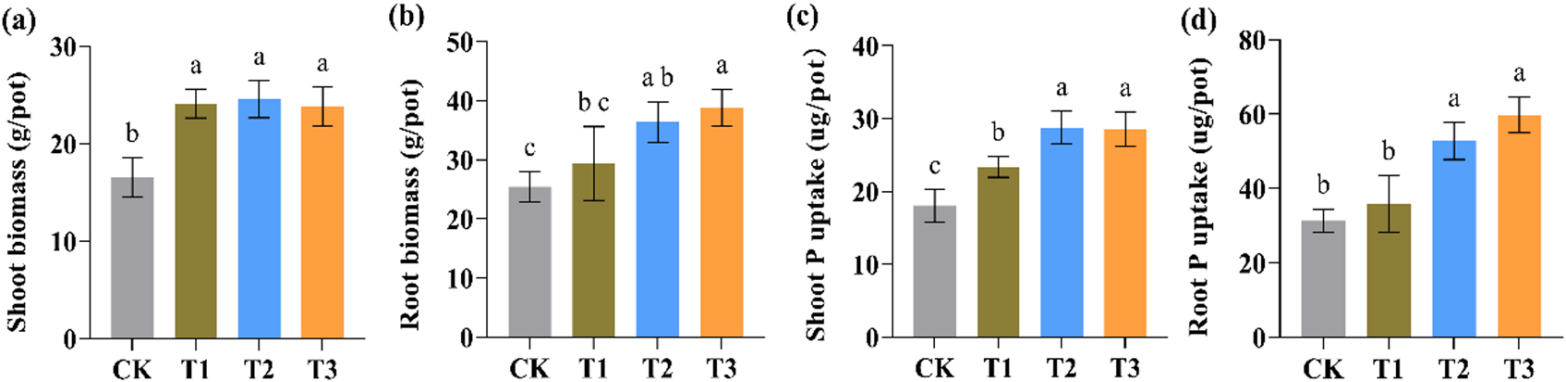
Effects of HA on biomass and P uptake of *S. miltiorrhiza*. Data represent mean ± standard error (SE, *n* = 4). Differences among treatments were analyzed by one-way ANOVA, and different lowercase letters indicate significant differences at *P* < 0.05.

Application of HA significantly enhanced biomass accumulation and P uptake in *S. miltiorrhiza* (Fig. 1). Shoot biomass increased by 45.56%, 48.43%, and 43.84% under T1, T2, and T3 treatments, respectively, compared to the CK (Fig. 1a, F = 20.89, *P* < 0.01). Similarly, Shoot P uptake rose significantly by 65.81% and 58.34% under T3 and T4 treatments (Fig. 1b, F = 29.17, *P* < 0.01). Root biomass also increased significantly by 43.02% and 52.59% under T2 and T3 treatments, respectively, compared to the CK (Fig. 1c, F = 11.43, *P* < 0.01). Similarly, root P uptake was enhanced by 68.59% and 91.05% under T2 and T3 treatments, respectively, compared to the CK (Fig. 1d, F = 31.79, *P* < 0.01).

### Effects of HA on physicochemical properties of rhizosphere soil

Application of HA significantly increased the contents of soil organic carbon (SOC), total nitrogen (TN), nitrate (NO_3_^-^-N), and ammonium (NH_4_^+^-N) in the rhizosphere soil of *S.miltiorrhiza* copmared to the CK(Table 1). Specifically, the SOC content under T2 and T3 treatments significantly increased by 3.64-fold and 1.66-fold, respectively (F = 48.05, *P* < 0.01). The TN content under T2 and T3 treatments was significantly elevated by 21.44% and 20.01%, respectively (F = 9.51, *P* < 0.01). The NH_4_^+^-N content also increased markedly by 89.13% and 57.68% under T2 and T3 treatments, respectively (F = 8.701, *P* < 0.01). In addition, the NO_3_^-^-N content in the T1 treatment significantly increased by 57.60% (F = 7.768, *P* < 0.01). In contrast, HA application significantly reduced both total phosphorus (TP) and Olsen-P contents. Compared to the CK, the TP content decreased by 14.62% and 25.42% under T2 and T3 treatments, respectively (F = 10.40, *P* < 0.01). The Olsen-P content under T2 treatment significantly decreased by 15.20% (F = 53.25, *P* < 0.001). Furthermore, HA application altered the phosphatase activity in the rhizosphere soil, with Acid phosphatase (ACP) activity significantly increased by 68.72% and 66.67% under T2 and T3 treatments, respectively, compared to CK (F = 15.50; *P* < 0.001).

**Table 1:** Effects of HA on rhizosphere soil properties and phosphatase activities of *S. miltiorrhiza*.

Treatment	CK	T1	T2	T3
SOC (g/kg)	6.33 ± 0.88d	45.23 ± 5.19a	29.44 ± 5.48b	16.85 ± 0.97c
TN (g/kg)	0.68 ± 0.02b	0.84 ± 0.02a	0.83 ± 0.05a	0.82 ± 0.05a
NO_3_^−^-N (mg/kg)	17.49 ± 2.54c	40.37 ± 9.54a	27.56 ± 2.84b	28.93 ± 2.81b
NH_4_^+^-N (mg/kg)	7.96 ± 1.23c	14.77 ± 2.81ab	15.07 ± 1.27a	12.56 ± 0.98b
TP (g/kg)	0.76 ± 0.03a	0.64 ± 0.03b	0.64 ± 0.03bc	0.57 ± 0.06c
Olesn-P (mg/kg)	11.05 ± 0.44a	10.72 ± 0.65ab	8.86 ± 0.38c	9.37 ± 0.61b
ALP (ug p-nitrophenol/g/h)	128.51 ± 3.16b	130.26 ± 2.45ab	135.72 ± 3.21a	129.68 ± 3.58ab
ACP (ug p-nitrophenol/g/h)	33.15 ± 5.55b	50.05 ± 3.15a	55.94 ± 2.73a	55.26 ± 5.89a

### Effects of HA on phosphorus pools and fractions in the rhizosphere soil

**Fig. 2:**
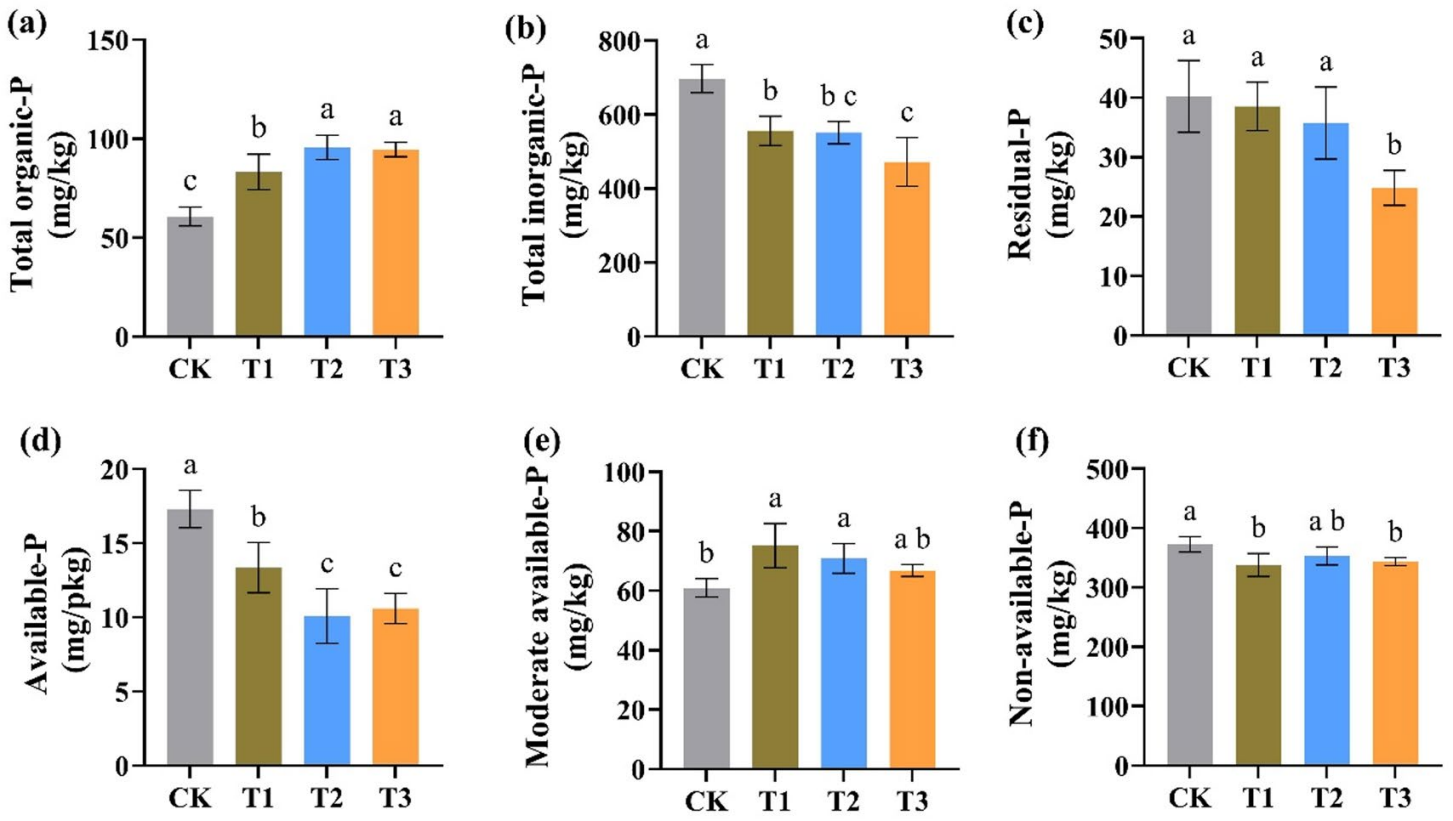
Effects of HA on the P pools in the rhizosphere soil of *S.miltiorrhiza*. Values shown are mean±standards error (SE; *n* = 4). Different lowercase letters in each column indicate significant differences (*P* < 0.05) among all treatments based on the one-way ANOVA followed by Tukey-HSD.

Application of HA significantly increased the total organic phosphorus content (Po) in the rhizosphere soil of *S.miltiorrhiza* by 57.41% and 55. 63% under T2 and T3 treatments, respectively, compared to the CK (Fig. 2a; F = 21.78, *P* < 0.01). In contrast, both total inorganic phosphorus (Pi) and residual-P contents were significantly reduced following HA application (Fig. 2b and c). The Pi content decreased by 20.91% and 32.29% under the T2 and T3 treatments, respectively, compared to CK (Fig. 2b, F = 16.23, *P* < 0.01); while residual-P declined by 38.23% under T3 treatment (Fig. 2c, F = 9.752, *P* < 0.05). In addition, HA also markedly affected P bioavailability. Compared to CK, the available-P content decreased by 48.70% and 44.37% under T2 and T3 treatments, respectively (Fig. 2d, F = 74.85, *P* < 0.01). Whereas moderate available-P increased by 29.63% and 18.47%, respectively (Fig. 2e, F = 14.28, *P* < 0.01). Non-available P decreased by 7.81% under T3 treatment (Fig. 2f, F = 5.534, *P* = 0.032).

**Fig. 3:**
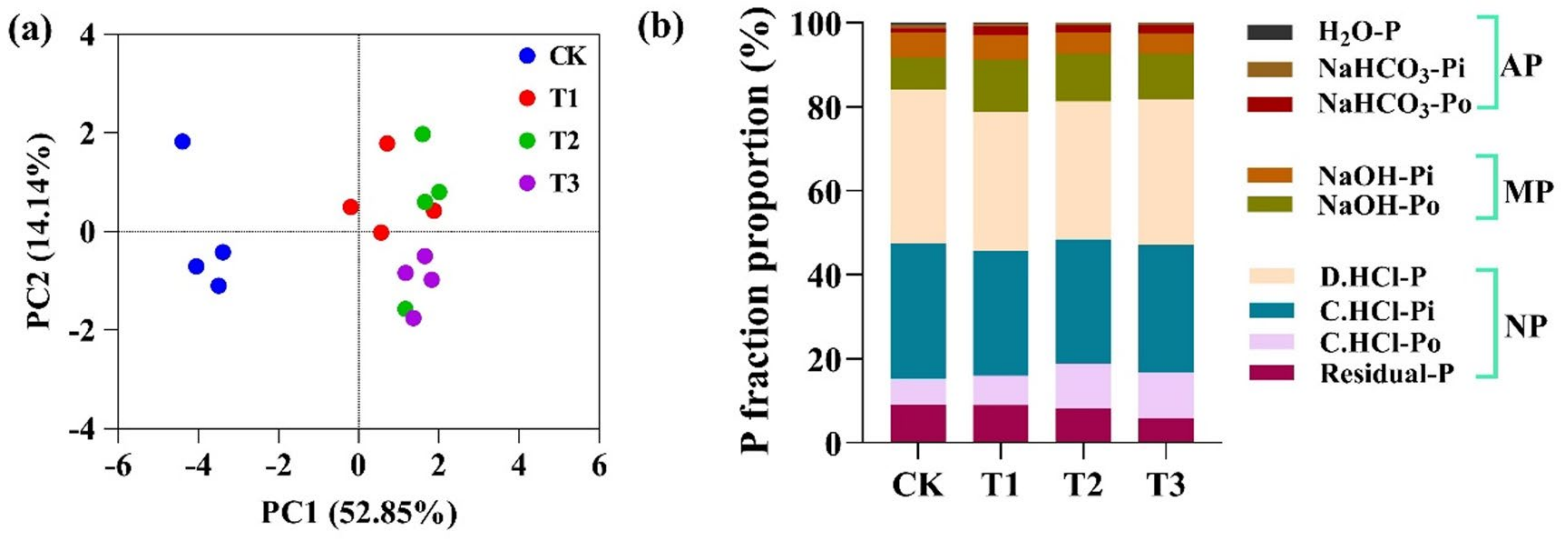
Changes in soil phosphorus (P) fractions under HA treatment. **(a)** Principal-component analysis (PCA) revealed distinct clustering of the P fractions across HA treatments; **(b)** Relative proportions (%) of P fractions under different HA treatments. NP: non-available P; MP: moderate available P; AP: available P.

**Fig. 4:**
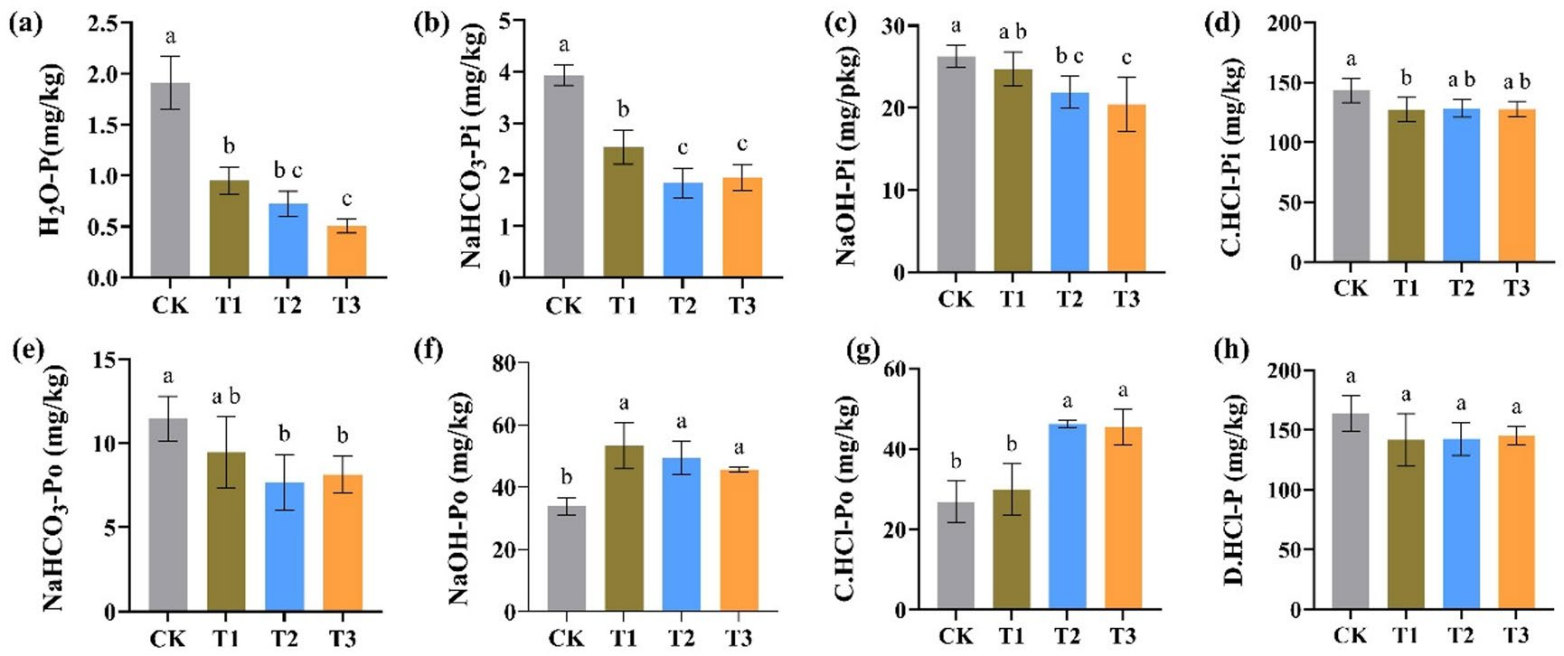
Effects of HA on P fractions in the rhizosphere soil of *S.miltiorrhiza*. Values shown are mean ± standard error (SE; *n* = 4). Different lowercase letters in each column indicate significant differences (*P* < 0.05) among all treatments based on the one-way ANOVA followed by Tukey-HSD.

Principal component analysis (PCA) exhibited a clear separation between HA-treated and control samples, with the first two axes accounting for 77.75% of the total variation (Fig. 3a). The rhizosphere soil was predominantly composed of low-available P forms, such as NaOH-P, D.HCl-P, C.HCl-P, and Residual-P, collectively representing 81.80% to 85.67% of the total P pool (Fig. 3b). HA application significantly reshaped the profile of individual P fractions (Fig. 4). Within the inorganic P (Pi) pools, H_2_O-P, NaHCO_3_-Pi, and NaOH-Pi were significantly reduced. Specially, compare to the CK, the H_2_O-P decreased by 62.1% and 73.61% undre T2 and T3 treatments, respectively (Fig. 4a, F = 75.05, *P* < 0.001), The NaHCO_3_-Pi contents decreased by 53.21% and 50.48%, respectively (Fig. 4b, F = 63.03, *P* < 0.001), the NaOH-Pi content was reduced by 22.23% under T3 treatment (Fig. 4c, F = 6.704, *P* = 0.003). Among organic P (Po) fractions, NaOH-Po and C.HCl-Po increased significantly, while NaHCO_3_-Po declined. Compare to CK, the NaHCO_3_-Po decreased by 44.92% and 37.40% under T2 and T3 treatments (Fig. 4e, F = 8.609, *P* = 0.012), the NaOH-Po increased by 57.11% and 45.67%, respectively (Fig. 4f, F = 15.21, *P* < 0.001), and C.HCl-Po increased by 72.27% and 69.34% under T2 and T3 treatments, respectively (Fig. 4g, F = 23.50, *P* < 0.001).

### Correlation of soil properties related to P fractions

**Fig. 5:**
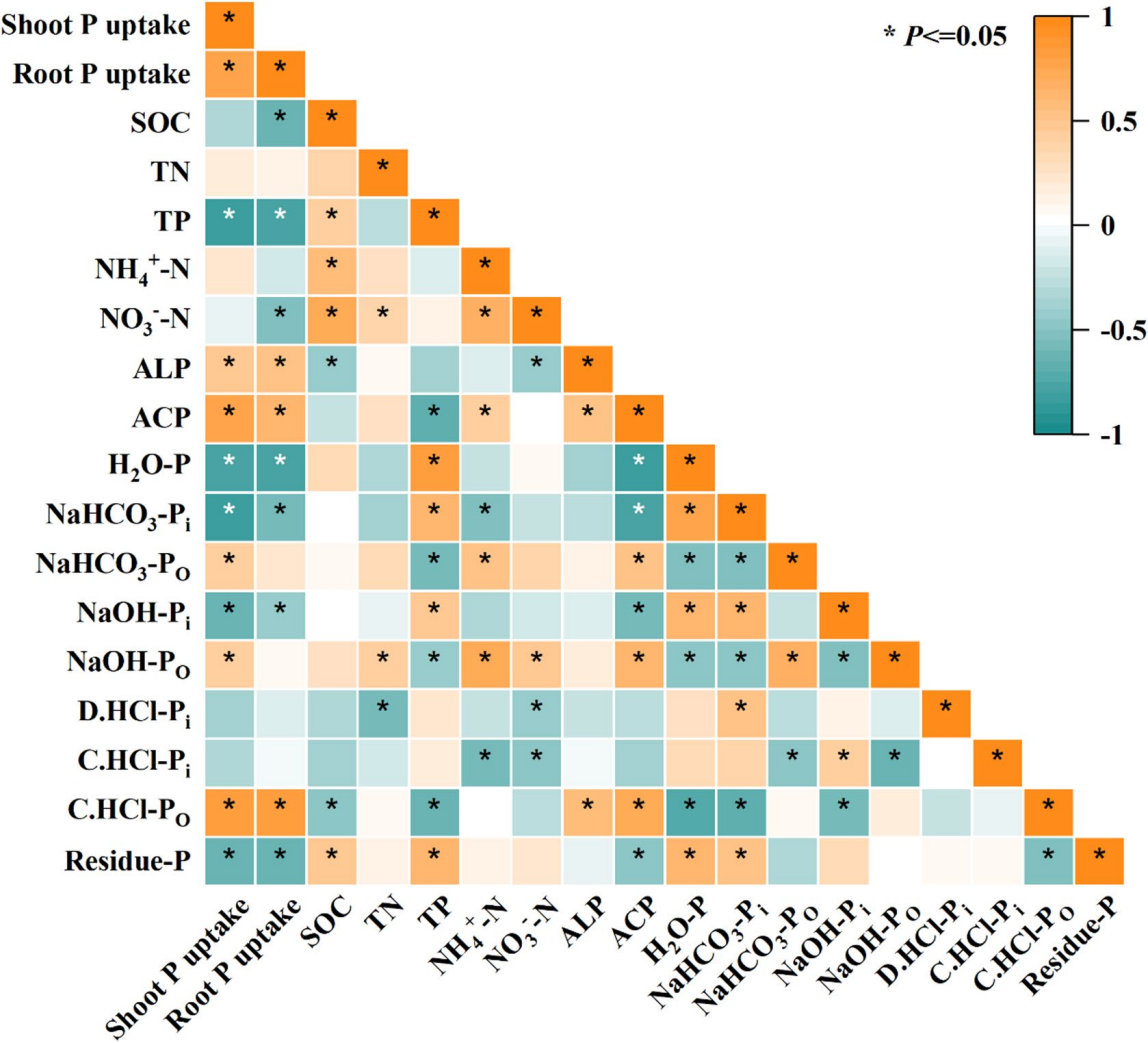
Pearson correlation heatmap depicting relationships among P uptake, P fractions, and soil properties of *S.miltiorrhiza*. An asterisk (*) indicates significant correlations at *P* < 0.05.

Correlation analysis further revealed that shoot P uptake, root P uptake, and acid phosphatase (ACP) activity were major factors driving these changes (Fig. 5). Shoot P uptake was significantly negatively correlated with inorganic P, such as H_2_O-Pi, NaHCO_3_-Pi, and NaOH-Pi (*ρ* = -0.793, -0.813, and − 0.603, respectively, *P* < 0.05), and positively correlated with ALP and ACP activity (*ρ* = 0.485 and 0.766, *P* < 0.05). Root P uptake showed a significantly negative correlation with SOC, TP, NO_3_^-^-N, H_2_O-P, NaHCO_3_-Pi, and NaOH-Pi (*ρ* = -0.612, -0.783, -0.507, -0.783, -0.598, and − 0.411, respectively, *P* < 0.05), and positive correlations with the ALP and ACP (*ρ* = 0.517 and 0.616, respectively, *P* < 0.05). The ACP activity was positively correlated with organic P fractions, such as NaHCO_3_-Po, NaOH-Po, and C.HCl-Po (*ρ* = 0.613 and 0.727, respectively, *P* < 0.05), and negatively correlated with inorganic P, such as H_2_O-Pi, NaHCO_3_-Pi, and NaOH-Pi (*ρ* = -0.836, -0.799, and − 0.574, respectively, *P* < 0.05). The NaHCO_3_-Pi showed a significantly negative correlation with NH_4_^+^-N (*ρ* = -0.524, *P* < 0.05), and the D.HCl-Pi and C.HCl-Pi showed a significantly negative correlation with NO_3_^-^-N (*ρ* = -0.401 and − 0.463, respectively, *P* < 0.05).

### Effects of HA on the composition and microbial biodiversity involved in P metabolism

**Fig. 6:**
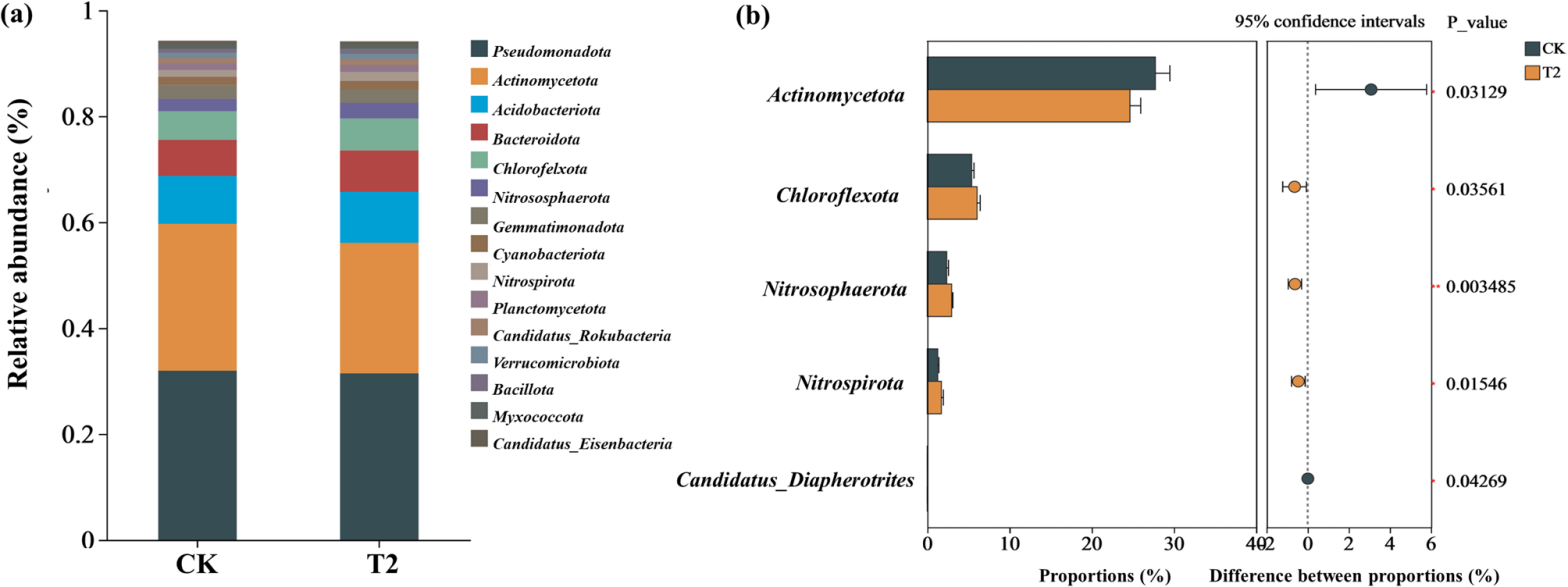
Composition and biodiversity of rhizosphere microbial communities associated with P cycling at the phylum level under different treatments. **(a)** The normalized relative abundance (Reads Per Kilobase Million, RPKM) of the top 15 P-cycling microbial phyla. **(b)** Differences in the relative abundance (RPKM) of microbial phyla. The bar plot displays the mean proportions (% of total RPKM) of differential microorganisms. Significant differences between groups are indicated at *P* < 0.05 (two-tailed Wilcoxon test, FDR-adjusted).

**Fig. 7:**
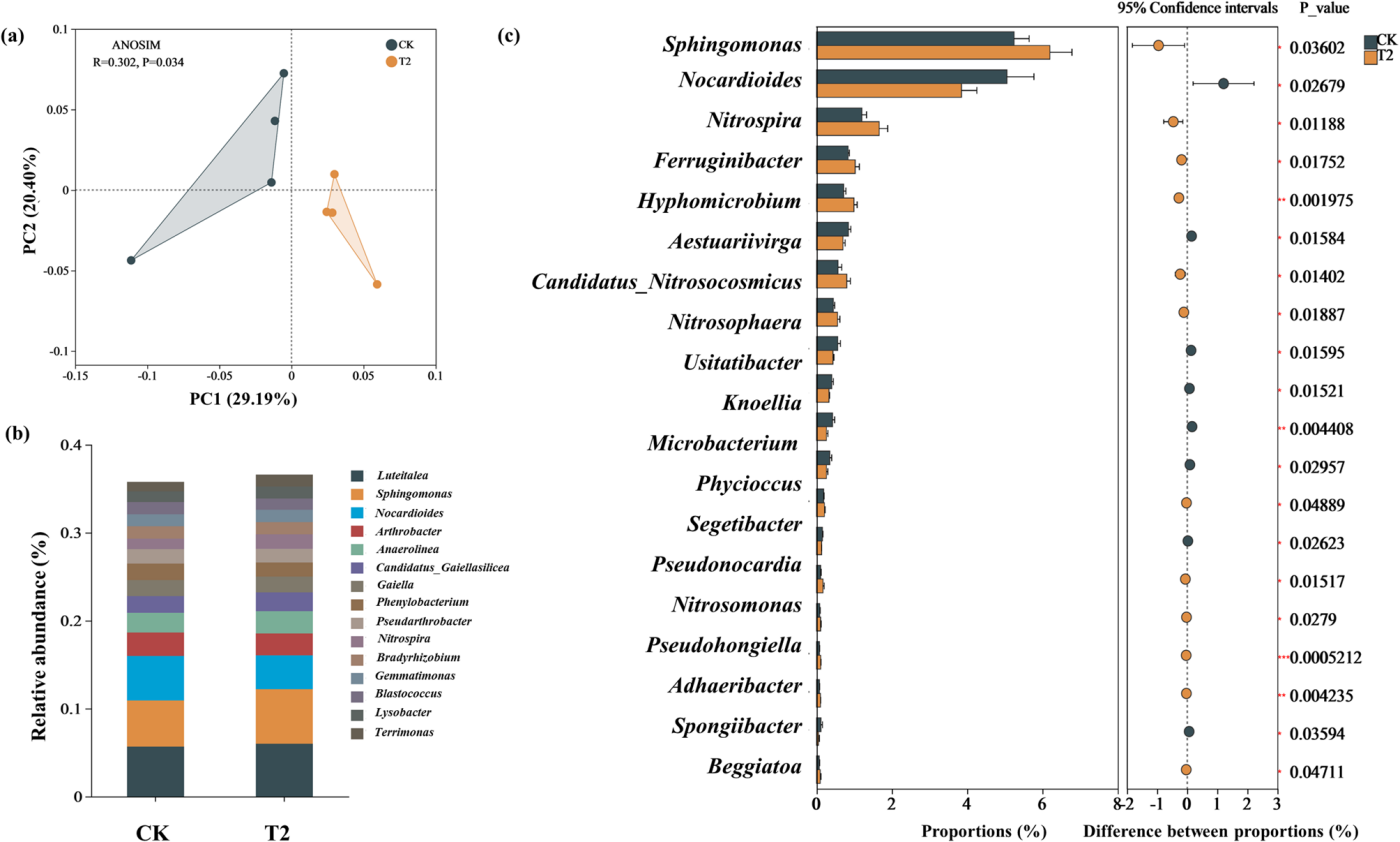
Composition and biodiversity of rhizosphere microbial communities associated with P cycling at the genus level under CK and T2 treatments. **(a)** Beta diversity illustrated by PCoA based on the Bray-Curtis distances; **(b)** The normalized relative abundance (Reads Per Kilobase Million, RPKM) of the top 15 P-cycling microbial genera. **(c)** Differences in the relative abundance (RPKM) of microbial genera. The bar plot displays the mean proportions (% of total RPKM) of differential microorganisms. Significant differences between groups are indicated at *P* < 0.05 (two-tailed Wilcoxon test, FDR-adjusted).

To elucidate the mechanism by which humic acid (HA) influences P transformation in rhizosphere soil, we compared the composition and diversity of P-cycling microbes between the control (CK) and HA-treated (T2) groups using metagenome sequencing. A total of 8 samples were sequenced, yielding approximately 54.79GB of clean reads, ranging from 69.10 to 79.53 million high-quality reads per sample. A non-redundant gene catalog comprising 3,596,916 genes (average length 400.39 bp) (Fig. 6a) was constructed. Sequencing Coverage across samples ranged from 99.12% to 99.45%. Principal coordinate analysis (PCoA) combined with ANOSIM, based on Bray-Curtis distance, revealed significant differences in P-cycling microbial communities between T2 and CK (ANOSIM, *r* = 0.302, *P* = 0.034), with the first two axes explaining 65.52% of the total variation (Fig. 7). The microbial communities were dominated at the phylum level by *Pseudomonadota*, *Actinomycota*, *Acidobacteriota*, *Bacteroidota*, *Chloroflexota*, and *Nitrososphaerota* (Fig. 6a). At the genus level, the top 10 genera included *Luteitalea*, *Sphingomonas*, *Nocardioides*, *Arthrobacter*, *Anaerolinea*, *Gaiella*, *Phenylobacterium*, *Pseudarthrobacter*, *Nitrospira*, and *Bradyrhizobium* (Fig. 7b). Differential abundance analysis using the two-tailed Wilcoxon test indicated significant enrichment of *Chloroflexota*, *Nitrososphaerota*, and *Nitorspirota* in the T2 group, while *Actinomycota* was enriched in the CK group at the phylum level (Fig. 6b). At the genus level, a total of 97 genera were differentially abundant, with 50 enriched in the T2 group, such as *Sphingomonas*, *Nitrospira*, *Ferruginibacter*, *Hyphomicrobium*, and *Nitrosophacera*, and 47 enriched in CK, such as *Nocardioides*, *Aestuariivirga*, *Usitatbacter*, and *Knoellia* (Fig. 7c).

## Discussion

The present study demonstrated that application of humic acid (HA) significantly promoted the growth and phosphorus (P) uptake of *S.miltiorrhiza*, concurrent with shifts in soil P fractions and microbial P-cycling processes. These finding suggest that HA application may enhance P bioavailability and is associated with shifts in the functional structure of microbial communities potentially involved in soil P mobilization and plant P utilization.

### HA effects on plant growth and P uptake

Humic acid (HA) functions as a bio-stimulant that enhances nutrient uptake and promotes plant growth^32^. Previous studies have suggested that HA may act by activating auxin-related signaling pathways and modulating hormone-related gene expression or metabolism (e.g., increasing IAA content), which could in turn stimulate root development and support overall plant growth^33^^, 34^. In line with these observations, our results showed that HA application increased root biomass accumulation of *S. miltiorrhiza*, possibly through enhanced root elongation linked to hormonal pathways (Fig. 1). Although phosphorus in plants is primarily derived from soil, its bioavailability is often limited due to strong fixation by soil components, which can reduce the efficiency of P fertilizers and constrain plant productivity^35^. Humic acid, as a natural organic macromolecule containing functional groups such as carboxylic (-COOH) and phenolic (-OH), is known to reduce P fixation and enhance P availability, potentially chelating metal ions such as calcium, iron, and aluminum^36^. For instance, in calcareous soils, HA has been reported to convert insoluble P (e, g., Ca_10_-P) into more plant-available forms (e.g., Ca_2_-P), thereby increasing available P content^37^. Consistent with this mechanism, our results showed that the T2 and T3 treatments increased P uptake in *S. miltiorrhiza* shoot by 65.81% and 58.34%, and in the root by 68.59% and 91.05% compared to non-HA treatments (Fig. 1b and d). This increase is likely related to reduced P fixation and enhanced P availability, which may have facilitated greater P uptake and accumulation in plants^38^. The observed decrease in rhizosphere soil Olsen-P content following HA application likely reflects multiple interacting processes, including enhanced plant P uptake, microbial immobilization, and potentially chemical stabilization by HA.

### HA on soil P transformation and bioavailability

Plants and microorganisms play a central role in converting inorganic phosphorus (Pi) into organic forms (Po), representing a key pathway for Po accumulation in soils^39^. In this study, HA application was associated with increased Po contents in the rhizosphere of *S. miltiorrhiza* (Fig. 2), suggesting a potential link with HA-induced stimulation of root exudation, such as sugars and amino acids, which may supply bioavailable carbon to rhizosphere microbial communities, thereby fostering microbial activity and residue accumulation^40,41^. The observed increases in Po were accompanied by significant changes in its composition: the increase in labile NaHCO_3_-Po was positively correlated with enhanced ACP activity (Figs. 3c and 5), indicating a potential association between microbial activity and Po mineralization dynamics of this labile organic P pool. This process may be further facilitated by the HA-induced increase in soil organic carbon (SOC) content (Table 1). In contrast, the more stable Po fractions, NaOH-Po and C.HCl-Po, also increased (Fig. 4e and h), potentially reflecting greater inputs of root-derived organic materials and microbial residues under HA treatment. In addition to these pant-mediated effects, HA may directly influence microbial activity by providing a favorable micro-environment or serving as a source of trace elements and organic compounds that modulate microbial metabolism. Such direct interactions could further shape the composition and function of P-cycling microbial communities, complementing the indirect pathways discussed above.

Plant P use efficiency is strongly influenced by the mobilization of soluble P and the mineralization of Po^42^. Consistent with this, the HA application significantly increased phosphatase activity in the rhizosphere soil (Table 1), corroborating earlier reporting that HA enhances ACP activity and facilitates the conversion of Po into readily available Pi forms^43,44^. This enzymatic enhancement appears to contribute to both organic P mineralization and solubilization of insoluble Pi, coinciding with an increase in proportions of labile and moderately labile P fractions and a reduction in sparingly soluble P (Fig. 2d-f). As a result of the enhanced phosphatase activity and the shifts in P fractions, the Pi pool was markedly affected. HA treatments were associated with enhanced biomass and P uptake in *S. miltiorrhiza* (Fig. 1), alongside a significant decrease in total Pi compared to the control (Fig. 2b). Specifically, the highly soluble H_2_O-P fraction (directly available to plants) and the dynamic reservoir comprising NaHCO_3_-Pi (readily desorbable P) and NaOH-Pi (Fe/Al-bound P) were depleted under conditions of enhanced plant uptake^45^, consistent with HA-induced increase in P bioavailability. Collectively, these observations suggest that HA may influence P availability through a combination of direct effects on enzyme activity and indirect effects mediated by shifts in soil physicochemical and biological properties.

### HA on soil microorganisms related to P cycling

Metagenomic analysis revealed that HA application restructured the P-cycling microbial communities, resulting in a significant divergence between HA-treated (T2) and CK soils (Fig. 7a). At the genus level, the enrichment of *Sphingomonas*,* Ferruginibacter*, *Nitrospira*, and *Nitrososphaera* in HA-treated soils points to a shift toward taxa possessing diverse P-acquisition strategies (Fig. 7c). Many *Sphingomonas* species have been reported to promote plant growth and enhance stress resistance, owing to their functional capabilities such as phosphate solubilization and phytohormone production^46^. Studies have further indicated a positive correlation between the relative abundance of *Sphingomonas* and of Olsen-P content, suggesting that application of microbial amendments may improve the soil nutrient environment by increasing both available nutrients and the abundance of beneficial microorganisms^47^. Phosphorus deficiency in the soil is conventionally addressed through P fertilizer application; however, P anions in chemical fertilizer are highly reactive and can be quickly fixed through interactions with Fe^3+^ in soil, rendering much of the applied P unavailable to plants. Some studies suggest that *Ferruginibacter* may be involved in P release from Fe-P complexes, potentially through mechanisms such as reduction of ferric ion (Fe^3+^) to soluble ferrous iron (Fe^2+^)^48^. Microbial N and P cycling processes are closely coupled. During nitrification, nitrifiers such as *Nitrospira* and *Nitrososphaera* may produce organic acids (e.g., citric and oxalic acid), which may lower rhizosphere pH and facilitate the dissolution of insoluble Pi compounds (e.g., Ca-P, Al-P, and Fe-P)^49–51^. This acidic environment may further promote synergistic interactions with P-solubilizing microorganisms (PSMs), establishing a coupled “nitrification-P solubilization” effect that could improve P availability^52^. Notably, Certain PSMs are known to contribute primarily via proton release driven by NH₄⁺ assimilation. Consistent with these potential mechanisms, our results show that HA application increased the abundance of nitrifying microorganisms, including *Nitrospira* and *Nitrososphaera*. This, along with the observed negative correlations between NH_4_^+^-N and labile P pools, is consistent with potential synergy between nitrification and P solubilization (Fig. 5). These findings indicate a potential association between HA application and enhanced nutrient use efficiency, possibly mediated by microbial coupling processes, highlighting its consideration in sustainable agriculture.

In this study, shifts in the relative abundance of microbial taxa involved in P cycling were observed following HA application. The NR-based analysis provided taxonomic-level resolution; the metabolic pathways potentially involved in P transformations remain to be explored through functional databases such as KEGG, eggNOG, or CAZy. In addition, the present findings were obtained under controlled pot conditions and may not readily extend to field-scale systems. To this end, gradient trials anchored to the optimal application rate identified here would help assess how these effects manifest under field conditions.

## Conclusion

This study provides evidence that humic acid (HA) application is associated with enhances phosphorus (P) acquisition in *S. miltiorrhiza*, accompanied by shifts in the microbiome and increased indicators of microbial-mediated P solubilization. Humic acid application significantly increased plant P uptake while reducing soil Olsen-P content, suggesting enhanced P mobilization from the soil to the plant. The shift was accompanied by a transformation of soil fractions from inorganic to organic forms and a pronounced increase in acid phosphatase activity, suggesting HA-induced stimulation of microbial activity. Comparative high-throughput sequencing between T2 and control groups revealed that HA enriched key microbial genera involved in P-cycling, including *Sphingomonas*, *Nitrospira*, and *Ferruginibacter*. Collectively, these findings suggest that HA may improve P bioavailability through microbially mediated processes, pointing to a potential approach to improve P use efficiency in agricultural systems. Further field-based studies are needed assess whether these findings hold under continuous-cropping *S. militiorrhiza* systems and to evaluate their applicability to other soils with similar P limitations.

## Materials and methods

### Experimental design and treatments

The experiment was conducted from April to September 2024 on the campus of Shangluo College, Shaanxi Province. The site is located in the southern foothills of the Qinling Mountains (33°52′N, 109°56′E, elevation 850 m), a region renowned for producing high-quality *S. Miltiorrhiza* and recognized as a National Geographical Indication (GI) Product. In this area, *S. Miltiorrhiza* is conventionally cultivated for 2–3 years before harvest. Soil used in the pot experiment was collected from a field that had undergone three consecutive years of *S.miltiorrhiza* monocropping. The soil exhibited the following characteristics: soil organic carbon (SOC) 6.04 g/kg, total nitrogen (TN) 0.53 g/kg, total phosphorus (TP) 0.79 mg/kg, available phosphorus (Olsen-P) 12.73 mg/kg, and pH 8.3 (measured in 1:5 soil-water suspension). Liquid humic acid (HA) fertilizer (≥ 30 g/L free humic acid) was provided by the Humic Acid Ecological Engineering Research Centre of the University of Science and Technology. The humic acid in this fertilizer is derived from mineral sources (extracted from weathered coal). The test materials were the one-year-old seedlings of *S. Miltiorrhiza* provided by Shaanxi TASLY Plant Medicine Co., Ltd.

The HA concentration treatments were directly selected from our established protocol^53^, in which high HA concentrations (e.g., 50-fold dilution) were found to exert no significant stimulatory effect on S. miltiorrhiza growth and, in some cases, even inhibited root development. Accordingly, we designed a gradient comprising a potential inhibitory concentration (T1: 100-fold dilution), the recommended field application rate (T2: 200-fold dilution), and a lower concentration (T3: 400-fold dilution) to comprehensively evaluate the dose-dependent effects of HA on soil phosphorus dynamics and plant response. The treatments included: (I) CK: basal fertilization only (no HA); (II) T1: Basal fertilization + 100-fold diluted HA solution; (III) T2: Basal fertilization + 200-fold diluted HA solution; (IV) T3: Basal fertilization + 400-fold diluted HA solution. The basal fertilization, potting. Seedling transplantation and thinning procedures were performed as described previously^53^. In brief, a compound fertilizer (N-P_2_O_5_-K_2_O = 15-15-15) was applied at a rate of 1.5 g per pot and thoroughly mixed with the soil before being packed into pots (22 cm diameter $\times$ 30 cm height, 4.5 kg soil per pot). Five uniform seedlings were transplanted per pot and thinned to three after establishment. Thirty days after emergence, each pot received 500 mL of its respective HA dilution (CK pots received distilled water). The experiment employed a completely randomized design with four biological replications per treatment.

### Soil physicochemical parameters and soil enzyme activity assay

At harvest, *S.miltiorrhiza* plants were separated into stems, leaves, and roots. The tissues were oven-dried (105℃ for 30 min deactivation, then 75℃ to a constant weight) to determine the dry weight of aboveground and root biomass. Phosphorus content in ground tissue (∼0.2 g) was digested using the H_2_SO_4_-H_2_O_2_ method and determined using the molybdenum antimony colorimetric method. Plant P uptake was calculated as the product of the tissue P content and its biomass.

Rhizosphere soil from each pot was sieved (2 mm) and divided into two subsamples: one stored at -80 °C for soil enzyme activities and metagenomic analyses, and the other air-dried for physicochemical characterization. Soil organic carbon (SOC) was determined by potassium dichromate oxidation. Total nitrogen (TN) by the Kjeldahl nitrogen method. Ammonium (NH_4_^+^-N) and nitrate (NO_3_^−^-N) were extracted with 1 M KCl and subsequently analyzed using a flow injection analyzer. Total phosphorus (TP) and Olsen-P were determined using the H_2_SO_4_-molybdenum antimony resistance method and the NaHCO_3_-molybdenum antimony resistance method, respectively. Alkaline phosphate (ALP) and acid phosphate (ACP) activities were determined using the p-nitrophenylphosphate (PNPP) method. Soil P fractions were determined by the Tiessen-Moir sequential extraction method^54^. Seven fractions were obtained by successive extraction with H_2_O, 0.5 mol/L NaHCO_3_, 0.1 mol/L NaOH, 1 mol/L dilute HCl (D.HCl), and concentrated HCl (C.HCl). These were categorized into three major categories: organic phosphorus (Po), inorganic phosphorus (Pi), and residual phosphorus (Residual-P). The Po fraction includes extractable organic forms from NaHCO_3_ (NaHCO_3_-Po), NaOH (NaOH-Po), and concentrated HCl (C.HCl-Po). The Pi comprised inorganic extracts from NaHCO_3_ (NaHCO_3_-Pi), NaOH (NaOH-Pi), dilute HCl (D.HCl-Pi), and concentrated HCl (C.HCl-Pi). Based on mobility and bioavailability, phosphorus pools were classified into three categories: available phosphorus (H_2_O-P, NaHCO_3_-P), moderately available phosphorus (NaOH-P), and Non-available phosphorus (HCl-P, Residual-P)^55^.

### DNA extraction and metagenome sequencing analysis of rhizosphere soil

To further explore the microbiological mechanisms underlying the HA-induced improvement in P acquisition in *S. miltiorrhiza*, the T2 group, which exhibited pronounced shifts in soil P fractions together with enhanced plant P uptake, was selected along with the control (CK) for subsequent metagenomic analysis. For each analysis, total genome DNA was extracted from approximately 0.2 g rhizosphere soil using the E.Z.N.A. soil DNA Kit (Omega Bio-tek, Norcross, GA, USA). DNA concentration and quality were assessed using a NanoDrop2000 spectrophotometer and 1% agarose gel electrophoresis. The DNA was then fragmented to an average size of approximately 350 bp with a Covaris M220 focused-ultrasonicator (Gene Company Limited, China) for paired-end library construction. Sequencing was performed on Illumina NovaSeq X Plus (Illumina Inc., San Diego, CA, USA) at Majorbio Bio-pharm Technology Co., Ltd (Shanghai, China).

Raw sequencing reads were processed using Fastp (v0.23.0) to remove adapters and low-quality sequences (length < 50 bp or average quality value ≤ 20)^56^, generating approximately 69.10-79.53 million clean reads per sample. Clean reads from each sample were subjected to de novo assembly with MEGAHIT (v1.1.2)^57^, resulting in a total of 6,341,316 contigs. Contigs with a length of ≥ 300 bp were retained for subsequent gene prediction and annotation. Open reading frames (ORFs) were identified from the assembled contigs using Prodigal (v2.6.3), yielding 7,599,080 ORFs with a minimum length of 100 bp. These sequences were then clustered with CD-HIT (v4.6.1) using an identity threshold of ≥ 0.9 and a coverage threshold of ≥ 0.9, yielding a non-redundant gene catalog comprising 3,596,916 genes, with an average length of 400.39 bp^58^. Taxonomic annotation was carried out by aligning the representative nucleotide sequences against the Non-redundant (NR) protein database using Diamond (v2.0.13) with default parameters (e-value < 1 1 0^− 5)59^.

### Statistical analysis

The effects of humic acid on biomass, P uptake, and soil physicochemical properties were evaluated using one-way analysis of variance (ANOVA). Statistical analyses were performed using GraphPad Prism (version 10.4.1, GraphPad Software, CA, USA, https://www.graphpad.com). Beta diversity of microbial communities related to P metabolism was assessed using principal coordinates analysis (PCoA) based on Bray-Curtis distance. Analysis of similarities (ANOSIM) with 999 permutations was applied to test for a significant difference between the control (CK) and humic acid (HA) treatment. Differential microbial taxa associated with P metabolism were identified using the Majorbio Cloud platform (www.majorbio.com). Significant differences between groups are indicated at *P* < 0.05 using a two-tailed Wilcoxon test with false discovery rate (FDR) adjustment.

## Data Availability

The raw metagenomic sequencing data generated in this study have been deposited in the NCBI Sequence Read Archive (SRA) under the accession number PRJNA1337597 (https://www.ncbi.nlm.nih, gov/bioproject/PRHNA1337597). Other datasets used and/or analyzed during the current study are available from the corresponding author upon reasonable request.

## References

[1] Su, C. Y., Ming, Q. L., Rahman, K., Han, T. & Qin, L. P. *Salvia miltiorrhiza*: Traditional medicinal uses, chemistry, and pharmacology. *Chin. J. Nat. Med.* **13**, 163–182 (2015). 25835361 10.1016/S1875-5364(15)30002-9

[2] Chen, M. L. et al. Inoculation with *Glomus mosseae* improves the growth and salvianolic acid B accumulation of continuously cropped *Salvia miltiorrhiza*. *Appl. Sci.* **7**, 692 (2017).

[3] Jiang, Z. Q., Gao, W. & Huang, L. Q. Tanshinones, critical pharmacological components in *Salvia miltiorrhiza*. *Front. Pharmacol.* **10**, 202 (2019). 30923500 10.3389/fphar.2019.00202 PMC6426754

[4] Wu, H. M. & Lin, W. X. A commentary and development perspective on the consecutive monoculture problems of medicinal plants. *Chin. J. Eco-Agric*. **28**, 775–793 (2020).

[5] Zhang, H. Q. et al. Effect of fertilization regimes on continuous cropping growth constraints in watermelon is associated with abundance of key ecological clusters in the rhizosphere. *Agric*. *Ecosyst. Environ.* **339**, 108135 (2022).

[6] Wang, Y. et al. Variation in microbial community structure in the rhizosphere soil of *Salvia miltiorrhiza* Bunge under three cropping modes. *Acta Ecol. Sin*. **39**, 4832–4843 (2019).

[7] Li, H. et al. Soil fumigation with ammonium bicarbonate or metam sodium under high temperature alleviates continuous cropping-induced Fusarium wilt in watermelon. *Sci. Hortic.* **246**, 979986 (2019).

[8] Alewell, C. et al. Global phosphorus shortage will be aggravated by soil erosion. *Nat. Commun.* **11**, 4546 (2020). 32917863 10.1038/s41467-020-18326-7 PMC7486398

[9] Maathuis, F. J. M. Physiological functions of mineral macronutrients. *Curr. Opin. Plant. Biol.* **12**, 250–258 (2009). 19473870 10.1016/j.pbi.2009.04.003

[10] Xia, G. H. et al. Effects of different nitrogen and phosphorus ratios on growth and active components accumulation in *Salvia miltiorrhiza*. *Chin. J. Exp. Tradit Med. Form.* **22**, 1–6 (2016). 10.4268/cjcmm20162215 28933085

[11] Han, J. P. & Liang, Z. S. Regulation of *Salvia miltiorrhizae* growth and danshensu and tanshion Ⅱ_A accumulation under nitrogen and phosphorus. *Chin Herb Med*. **5**, 756–759 (2005).

[12] Laliberté, E., Lambers, H., Burgess, T. I. & Wright, S. J. Phosphorus limitation, soil-borne pathogens and the coexistence of plant species in hyperdiverse forests and shrublands. *New. Phytol*. **206**, 507–521 (2015). 25494682 10.1111/nph.13203

[13] Cao, Y. F. et al. Phosphorus availability influences disease-suppressive soil microbiome through plant-microbe interactions. *Microbiome* **12**, 185 (2024). 39342390 10.1186/s40168-024-01906-w PMC11439275

[14] Shen, J. B. et al. Phosphorus dynamics: from soil to plant. *Plant. Physiol.* **156**, 997–1005 (2011). 21571668 10.1104/pp.111.175232 PMC3135930

[15] Singh, L. M. & Chanu, C. K. Solubilization of phosphorus and potassium by soil microorganisms. *Just Agric.* **4**, 203 (2024).

[16] Maranguit, D., Guillaume, T. & Kuzyakov, Y. Land-use change affects phosphorus fractions in highly weathered tropical soils. *CATENA* **149**, 385–393 (2017).

[17] Liang, J. L. et al. Novel phosphate-solubilizing bacteria enhance soil phosphorus cycling following ecological restoration of land degraded by mining. *ISME J.* **14**, 1600–1613 (2020). 32203124 10.1038/s41396-020-0632-4 PMC7242446

[18] Zhang, Y. L., Li, Y., Wang, S. Z., Umbreen, S. & Zhou, C. F. Soil phosphorus fractionation and its association with soil phosphate-solubilizing bacteria in a chronosequence of vegetation restoration. *Ecol. Eng.* **164**, 106208 (2021).

[19] Alori, E. T., Glick, B. R. & Babalola, O. O. Microbial phosphorus solubilization and its potential for use in sustainable agriculture. *Front. Microbiol.* **8**, 971 (2017). 28626450 10.3389/fmicb.2017.00971 PMC5454063

[20] Wu, W. C. et al. Organic amendments promote soil phosphorus related functional genes and microbial phosphorus cycling. *Geoderma* **456**, 117247 (2025).

[21] Canellas, L. P. et al. Humic and fulvic acids as biostimulants in horticulture. *Sci. Hortic.* **196**, 15–27 (2015).

[22] Ampong, K., Thilakaranthna, M. S. & Gorim, L. Y. Understanding the Role of Humic Acids on Crop Performance and Soil Health. *Front. Agron.* **4**, 848621 (2022).

[23] Chen, Q. et al. Humic acid modulates growth, photosynthesis, hormone and osmolytes system of maize under drought conditions. *Agric. Water Manag*. **263**, 107447 (2022).

[24] Mora, V. et al. Action of humic acid on promotion of cucumber shoot growth involves nitrate-related changes associated with the root-to-shoot distribution of cytokinins, polyamines and mineral nutrients. J. *Plant. Physiol.* **167**, 633–642 (2010). 10.1016/j.jplph.2009.11.018 20185204

[25] Nardi, S., Ertani, A. & Francioso, O. Soil-root cross-talking: The role of humic substances. *J. Plant. Nutr. Soil. Sci.* **180**, 22–34 (2017).

[26] Lumactud, R. A., Gorim, L. Y. & Thilakarathna, M. S. Impacts of humic-based products on the microbial community structure and functions toward sustainable agriculture. *Front. Sustain. Food Syst.* **6**, 977121 (2022).

[27] Zhu, J., Li, M. & Whelan, M. Phosphorus activators contribute to legacy phosphorus availability in agricultural soils. *Rev. Sci. Total Environ.* **612**, 522–537 (2018). 10.1016/j.scitotenv.2017.08.095 28865270

[28] Regelink, I. C., Weng, L., Lair, G. J. & Comans, R. N. J. Adsorption of phosphate and organic matter on metal (hydr)oxides in arable and forest soil: a mechanistic modelling study. *Eur. J. Soil. Sci.* **66**, 867–875 (2015).

[29] Xiong, Q. Z. et al. The effective combination of humic acid phosphorus fertilizer regulating the form transformation of phosphorus and the chemical and microbial mechanisms of its phosphorus availability. *Agronomy* **13**, 1581 (2023).

[30] Maji, D., Misra, P., Singh, S. & Kalra, A. Humic acid rich vermicompost promotes plant growth by improving microbial community structure of soil as well as root nodulation and mycorrhizal colonization in the roots of Pisum sativum. *Appl. Soil. Ecol.* **110**, 97–108 (2017).

[31] Yang, L. et al. Research on Fusarium Wilt of *Salvia miltiorrhiza* and Its Pathogen. *China J. Chin Mater. Med*. **38**, 3987–3990 (2013). 24791484

[32] Zanin, L., Tomasi, N., Cesco, S., Varanini, Z. & Pinton, R. Humic Substances Contribute to Plant Iron Nutrition Acting as Chelators and Biostimulants. *Front. Plant. Sci.* **10**, 675 (2019). 31178884 10.3389/fpls.2019.00675 PMC6538904

[33] Zandonadi, D. B. et al. Humic acids as drivers of plant growth: regulating root development and photobiology through redox modulation. *Chem Biol Technol Agric*. **12**, 71 (2025).

[34] Santoro, V. et al. Phosphorus acquisition efficiency and transcriptomic changes in maize plants treated with two lignohumates. *Plants* **12**, 3291 (2023). 37765455 10.3390/plants12183291 PMC10535022

[35] Pang, F. et al. Soil phosphorus transformation and plant uptake driven by phosphate-solubilizing microorganisms. *Front. Microbiol.* **15**, 1383813 (2024). 38601943 10.3389/fmicb.2024.1383813 PMC11005474

[36] Jing, J. Y. et al. Effects of incorporating different proportions of humic acid into phosphate fertilizers on phosphorus migration and transformation in soil. *Agronomy* **13**, 1576 (2023).

[37] Liu, X. Q., Zhao, X. J. & Lv, J. L. Molecular characterization of size-fractionated humic acids derived from lignite and its activation of soil legacy phosphorus and Lactuca sativa growth-promoting performances. *ACS Omega*. **8**, 6838–6846 (2023). 36844549 10.1021/acsomega.2c07528 PMC9948213

[38] Yang, F. et al. Synthetic humic acids solubilize otherwise insoluble phosphates to improve soil fertility. *Angew Chem. Int. Ed. Engl.* **58**, 18813–18816 (2019). 31621138 10.1002/anie.201911060 PMC6973123

[39] Li, T. et al. Characteristics of organic phosphorus pool in soil of typical agriculture systems in South China. *Horticulturae* **8**, 1055–1069 (2022).

[40] Canarini, A., Kaiser, C., Merchant, A., Richter, A. & Wanek, W. Root exudation of primary metabolites: mechanisms and their roles in plant responses to environmental stimuli. *Front. Plant. Sci.* **10**, 157–176 (2019). 30881364 10.3389/fpls.2019.00157 PMC6407669

[41] Canellas, L. P., Olivares, F. L., Canellas, N. O., Mazzei, P. & Piccolo, A. Humic acids increase the maize seedlings exudation yield. *Chem. Biol. Technol. Agric.* **6**, 3–17 (2019).

[42] Meng, X. et al. Effects of irrigation, phosphate fertilizers, and arbuscular mycorrhizal fungal interaction on soil nutrient content in alfalfa fields. *Pratacult Sci.* **40**, 1220–1231 (2023).

[43] Shi, X. et al. Effect of exogenous organic matter on phosphorus forms in middle-high fertility cinnamon soil. *Plants* **13**, 1313–1328 (2024). 38794384 10.3390/plants13101313 PMC11125253

[44] Li, Y. et al. Humic acid fertilizer improved soil properties and soil microbial diversity of continuous cropping peanut: A three-year experiment. *Sci. Rep.* **9**, 12014–12025 (2019). 31427666 10.1038/s41598-019-48620-4 PMC6700118

[45] Yang, Z. X., Zhou, H. P., Xie, W. Y. & Liu, Z. P. Response of phosphorus components to phosphate surplus in cinnamon soil under long-term fertilization. J. *Plant. Nutr. Fertil.* **26**, 924–933 (2020).

[46] Kim, Y. J. et al. Comprehensive genome analysis on the novel species Sphingomonas panacis DCY99T reveals insights into iron tolerance of ginseng. *Int. J. Mol. Sci. 21*, 2019. (2020). 10.3390/ijms21062019 PMC7139845 32188055

[47] Asaf, S., Khan, A. L., Khan, M. A., Al-Harrasi, A. & Lee, I. J. Complete genome sequencing and analysis of endophytic Sphingomonas sp. LK11 and its potential in plant growth. 3 *Biotech 8*, 389–403. (2018). 10.1007/s13205-018-1403-z PMC6111035 30175026

[48] Li, M. J., Ye, X. X., Da, Y. M., Sun, Q. Y. & Zhou, G. W. Unveil of the role of fungal taxa in iron (Ⅲ) reduction in paddy soil. *Front. Microbiol.* **14**, 1334051–1334061 (2024). 38328582 10.3389/fmicb.2023.1334051 PMC10848163

[49] D’Acunto, L., Andrade, J. F., Poggio, S. L. & Semmartin, M. Diversifying crop rotation increased metabolic soil diversity and activity of the microbial community. *Agric. Ecosyst. Environ.* **257**, 159–164 (2018).

[50] Xu, H. R. et al. Plant-root microbiota interactions in nutrient utilization. *J. Front Agric. Sci. Eng*. **12**, 16–26 (2025).

[51] Tao, D. X. & Gao, Y. Z. Advances on the strategies of soil phosphate solubilizing microorganisms to promote plant phosphorus uptake. *Acta Ecol. Sin*. **43**, 4390–4399 (2023).

[52] Li, H. M., Wang, R., Zhong, Y. M., Shi, W. M. & Li, Y. L. Rhizosphere communication and its effects on improving phosphorus utilization in high-input vegetable production system: A review. *Acta Pedol. Sin*. **59**, 924–934 (2022).

[53] Yue, L. et al. Wang. Effects of HA on the growth of *Salvia miltiorrhiza* and Soil enzyme activity [J]. *J. Cold-Arid Agricultural Sci.* **4** (8), 760–765 (2025).

[54] Luo, L., Ye, H. Y., Zhang, D. H., Gu, J. D. & Deng, O. P. The dynamics of phosphorus fractions and the factors driving phosphorus cycle in Zoige Plateau peatland soil. *Chemosphere* **278**, 130501 (2021). 34126697 10.1016/j.chemosphere.2021.130501

[55] Mahmood, M. et al. Changes in phosphorus fractions in response to long-term nitrogen fertilization in the loess plateau of China. *Field Crops Res.* **270**, 108207 (2021).

[56] Dai, R. et al. Effects of dietary crude protein levels in the concentrate supplement after grazing on rumen microbiota and metabolites by using metagenomics and metabolomics in Jersey-yak. *Front. Microbiol.* **14**, 1124917 (2023). 37200912 10.3389/fmicb.2023.1124917 PMC10185794

[57] Jouffret, V. et al. Increasing the power of interpretation for soil metaproteomics data. *Microbiome* **9**, 195 (2021). 34587999 10.1186/s40168-021-01139-1 PMC8482631

[58] Fu, L., Niu, B., Zhu, Z., Wu, S. & Li, W. CD-HIT: accelerated for clustering the next-generation sequencing data. *Bioinformatics* **28**, 3150–3152 (2012). 23060610 10.1093/bioinformatics/bts565 PMC3516142

[59] Chai, M., Zhang, Y., Cui, K., Bi, Y. & Zhang, N. Metagenomics reveals the temporal dynamics of the rumen resistome and microbiome in goat kids. *Microbiome* **12**, 14 (2024). 38254181 10.1186/s40168-023-01733-5 PMC10801991

